# Effects of Taxifolin on Osteoclastogenesis *in vitro* and *in vivo*

**DOI:** 10.3389/fphar.2018.01286

**Published:** 2018-11-12

**Authors:** Cong Cai, Changyu Liu, Liming Zhao, Hui Liu, Weijin Li, Hanfeng Guan, Libo Zhao, Jun Xiao

**Affiliations:** Department of Orthopaedic Surgery, Tongji Hospital, Tongji Medical College, Huazhong University of Science and Technology, Wuhan, China

**Keywords:** taxifolin, osteoclast, osteoporosis, RANK, NF-κB

## Abstract

Osteoporosis is a highly prevalent disease which has been a major public health problem and considered to be associated with chronic low-grade systemic inflammation and oxidative damage. Taxifolin is a natural flavonoid and possesses many pharmacological activities including antioxidant and anti-inflammatory. Because flavonoids have been confirmed to fight osteoporosis and promote bone health, the aim of this study was to investigate the effects of taxifolin on the formation and function of osteoclast. In this study, we examined the effects of taxifolin on osteoclast using both *in vitro* and *in vivo* studies. Taxifolin suppressed the activation of nuclear factor-κB, C-Fos and mitogen-activated protein kinase, and also decreased osteoclast-specific genes expression, including *Trap, Mmp-9, Cathepsin K, C-Fos, Nfatc1*, and *Rank*. Taxifolin also prevented reactive oxygen species (ROS) production following RANKL stimulation. In addition, taxifolin alleviated ovariectomized-induced bone loss by repressing osteoclast activity and decreasing serum levels of tumor necrosis factor-α, interleukin-1β, interleukin-6 and receptor activator of nuclear factor-κB ligand (RANKL) *in vivo*. Our results indicated that taxifolin inhibits osteoclastogenesis via regulation of modulation of several RANKL signaling pathways. Therefore, taxifolin may be considered as a potential alternative therapeutic agent for treating osteoclast-related diseases.

## Introduction

Bone homeostasis is maintained by the mutual function of osteoclastic bone resorption and osteoblastic bone formation. The imbalance caused by excessive bone resorption will lead to various osteopathic diseases, such as osteoporosis and Paget’s disease (Feng and McDonald, 2011). Osteoclasts are the main type of bone-resorptive giant polykaryons differentiated from monocyte-macrophage lineage precursor cells upon stimulation by two crucial factors, macrophage-colony stimulating factor (M-CSF) and receptor activator of NF-κB (RANK) ligand (RANKL) (Novack, 2011). M-CSF is crucial factor for osteoclast precursor proliferation and survival which can promote RANK expression. The binding of RANKL and RANK activates the tumor necrosis factor receptor associated factor 6 (TRAF6). TRAF6 subsequently results in the downstream signaling activation of NF-κB, MAPKs (ERK, JNK, and p38), PI3K/AKT, C-Fos, activator protein 1 (AP-1), and nuclear factor and activator of transcription (NFATc1). Osteoporosis has been considered to be associated with chronic low-grade systemic inflammation and oxidative damage. Increased production of pro-inflammatory cytokines such as TNF-α, IL-6, and IL-1β is associated with osteoclastic bone resorption (Mundy, 2007). In addition, reactive oxygen species (ROS), generated after the binding of RANKL to its receptor, also promotes osteoclastogenesis through RANK signaling pathways including AKT, MAPK and NF-κB (Lee et al., 2005; Kim et al., 2006; Sasaki et al., 2009). ROS could act as the intracellular secondary messengers in RANKL-induced osteoclastogenesis signaling pathways, and osteoclast differentiation can be inhibited by scavenging ROS (Lee et al., 2005; Chen et al., 2016). All these pathways mentioned above play essential roles in osteoclastogenesis and osteoclast function, stimulating osteoclast formation and thus bone resorption will lead to osteoporosis.

Taxifolin is a common flavonoid. Flavonoids are a group of secondary metabolic compounds widely found in plants, as the key ingredients of the human diet, and they have been very popular because of their many health-promoting and disease-preventive effects (Winkel-Shirley, 2001; Salaritabar et al., 2017). Emerging evidence suggests that taxifolin exerts various pharmacological action, including antioxidant, anti-inflammatory, antiviral, antibacterial activities, anticancer, and neuroprotective activities (Dok-Go et al., 2003; Wang et al., 2006; Manigandan et al., 2015; Zhao et al., 2015; Galochkina et al., 2016; Bijak, 2017). Taxifolin can ameliorate oxidative damage by modulating NF-κB signaling pathway (Wang et al., 2006; Kim et al., 2017). Taxifolin is an effective chemopreventive agent capable of modulating inflammation, as it inhibits NF-κB by down-regulating the levels of regulatory metabolites such as TNF-α.

Recently, taxifolin has been reported to stimulate osteoblast differentiation in bone marrow mesenchymal stem cells by inhibiting the nucleus translocation of NF-κB (Wang et al., 2017). Taxifolin can also promote osteoblast differentiation in MC3T3-E1 cells and inhibit osteoclastogenesis in RAW264.7 cells (Satue et al., 2013). However, whether taxifolin prevents bone loss in osteopenic mice and the specific pathway involved that taxifolin inhibits osteoclastogenesis remains unknown. In this study, we investigate the effects and the underlying mechanism of taxifion on osteoclastogenesis *in vitro* and ovariectomy-induced osteoporosis *in vivo*.

## Materials and Methods

### Reagents

Taxifolin (HPLC ≥ 98%) was purchased from Sigma-Aldrich (Shanghai, China). Recombinant soluble human M-CSF and mouse receptor activator of nuclear factor-κ B ligand (RANKL) were obtained from PeproTech (Rocky Hill, NJ, United States). The MTT Cell Proliferation and Cytotoxicity Assay Kit was purchased from Boster (Wuhan, China). The following antibodies were purchased from Cell Signaling Technology (Beverly, MA, United States): ERK (#9102), phospho-ERK (#4377), JNK (#9258), phospho-JNK (#4668), P38 (#8690), phospho-P38 (#4511), IKKβ (#8943), phospho-IKKα/β (#2697), P65 (#8242), phospho-P65 (#3033), IκB-α (#4812), phospho-IκB-α (#2859), NFATc1 (#8032), and RANK (#4845). Anti- C-Fos was purchased from Abcam (Cambridge, MA, United States). Antibodies against Cathepsin K, MMP-9, and TRAP were obtained from Proteintech Group (Wuhan, Hubei, China) The NF-κB and AP-1 probe was purchased from Beyotime (Shanghai, China). The TRAP staining kit and all other reagents were purchased from Sigma-Aldrich.

### Animals and Experimental Design

Four-month-old female C57BL/6 mice (21 ± 1 g) were purchased from the Experimental Animal Center of Tongji Medical College (Wuhan, China) and used for sham or bilateral ovariectomy (OVX) operation. All procedures were approved by the Ethics Committee on Animal Experimentation of Tongji Medical College, Huazhong University of Science and Technology (Wuhan, China). All mice were housed at the animal care facility of Tongji Medical College at 25°C with 12-h light/dark cycles and were allowed free access to normal mice chow and water. They were randomly assigned to three groups (*n* = 12/group): a sham treated group, ovariectomized (OVX) mice treated with normal saline, and OVX mice treated with taxifolin (50 mg/kg/day) dissolved in normal saline (Sun et al., 2014). Sham operation was performed by identifying the bilateral ovaries and ovariectomy was performed by removing the bilateral ovaries, both incisions were made through a dorsal approach. After the procedure, mice were allowed to recover for 1 day, then mice were injected intraperitoneally with normal saline or taxifolin six times per week for 6 weeks. After 6 weeks of intervention, the mice were sacrificed for the subsequent experiments, we measured the uterus wet weight to validate the success of ovariectomy.

### Microcomputer Tomography Analysis

After removal of soft tissues, microcomputer tomography (μCT) (μ-CT50 Scanco Medical, Bassersdorf, Switzerland) was performed on the distal femur. Scans were taken with a source voltage of 80 kV and 80 μA source current with a voxel size of 10 μm. The bone structural parameters of bone mineral density (BMD), bone volume/tissue volume (BV/TV), trabecular number (Tb. N.), trabecular thickness (Tb. Th.), and trabecular separation (Tb. Sp.) were quantitatively analyzed with the built-in software of the μCT. The 3-dimensional bone structure image slices were reconstructed using the built-in software. Nomenclature and abbreviations of parameters follow the recommendations of the American Society of Bone and Mineral Research.

### Bone Histological Analysis

Femur samples were decalcified for 1 week with 10% tetrasodium-EDTA aqueous solution at 4°C. The paraffin-embedded femur sections (5 μm thick) were prepared with a microtome and processed for histological observation of the metaphysis below the primary spongiosa by H&E and TRAP staining. Histological measurements and images were taken under a microscope. Trabecular bone was revealed in H&E-stained sections, numbers of osteoclasts were counted in the sections with TRAP staining.

### Serum Biochemistry

Blood was collected via retro-orbital puncture and sera were collected after centrifugation at 4000 rpm for 15 min at 25°C. Serum levels of TRAP, TNF-α, IL-1β, IL-6, RANKL, and OPG were determined by an enzyme-linked immunosorbent assay (ELISA) kit (BD Biosciences, San Jose, CA, United States) according to the manufacturer’s instructions. TRAP, TNF-α, IL-1β, and IL-6 ELISA kits were from BD Biosciences (San Jose, CA, United States), RANKL and OPG ELISA kits were from Boster (Wuhan, China).

### Cell Culture and Treatment

RAW264.7, a murine monocytic cell line, was obtained from the Cell Bank of the Chinese Academy of Sciences (Shanghai, China) and maintained in Dulbecco’s Modified Eagle’s medium (DMEM) supplemented with 10% heat-inactivated fetal bovine serum, streptomycin (100 mg/ml) and penicillin (100 U/ml) in a cell cultured incubator at 37°C and 5% CO_2_. We isolated and cultured primary bone marrow mononuclear cells (BMMCs) from C57BL/6 mice as we described previously (Guan et al., 2015). To induce osteoclast formation, both BMMCs and RAW264.7 were treated with RANKL (50 ng/ml, R&D) and BMMCs culture medium was also supplemented with 25 ng/ml M-CSF (Guan et al., 2015). The culture medium was replaced every day.

### Cytotoxicity Assay

RAW264.7 cells (3 × 10^3^ cells/well) were seeded in 96-well plates for cytotoxicity assay. After 24 h, cells were treated with various concentrations of taxifolin. After 3 days, an LC_50_ curve was calculated by GraphPad Prism 5 according to MTT cytotoxicity assay results.

### TRAP Staining and TRAP Enzyme Activity Assay

To examine the effects of taxifolin on osteoclast formation in cultured BMMCs and RAW264.7 cells, besides RANKL (50 ng/ml) and/or M-CSF (25 ng/ml), cells were treated with taxifolin as indicated concentrations for 4 days (Satue et al., 2013). Osteoclasts were identified by tartrate-resistant acid phosphatase (TRAP) staining kit (Sigma-Aldrich) according to the manufacturer’s protocol. TRAP-positive multinucleated cells with three or more nuclei were identified as osteoclasts (Asagiri and Takayanagi, 2007). Cell images were taken using a digital camera attached to an EVOS FL Auto microscope (Life Technologies, United States). TRAP enzyme activity in cultured medium collected from BMMCs was measured with a TRAP Assay Kit (Sigma-Aldrich, Shanghai, China) following the manufacturer’s instructions. TRAP enzyme activity was quantified using a Synergy fluorescence plate reader at 405 nm on a colorimetric plate reader.

### Actin Ring Formation Assays and DAPI Staining

RAW264.7 cells were treated with RANKL (50 ng/ml) and different concentrations of taxifolin for 4 days to form osteoclasts. Next, cells were added with immunol staining fix solution (Beyotime, Shanghai, China) for 10 min, then the cells were permeabilized with immunol staining wash buffer (Beyotime) for 5 min and incubated with TRITC phalloidin (Sigma-Aldrich, St. Louis, MO, United States) at 25°C for 30 min to visualize F-actin. After treatment with actin ring staining, cells were washed four times with phosphate buffer saline followed by staining with DAPI (Boster) for 5 min. Images were obtained using fluorescence microscope.

### Bone Pit Formation by Osteoclasts

RAW264.7 cells were treated with RANKL (50 ng/ml) for 4 days to form osteoclasts. After 4 days, mature osteoclasts were collected and seeded onto Corning Osteo Assay Surface (Corning Incorporated Life Science, United States) in a multiple well plate in complete medium in the presence of RANKL (50 ng/ml) and different concentrations of taxifolin for 3 days. Then, the disks were washed with 5% sodium hypochlorite for 5 min, and images of resorption pits were taken through light microscopy and resorption area was quantified by image analysis (BIOQUANT Image Analysis, Nashville, TN, United States).

### Measurement of ROS Production

RAW264.7 cells were cultured on 12-well plates, after treatment with taxifolin for 36 h, cells were incubated for 30 min in presence of RANKL (50 ng/ml). Subsequently, ROS production was measured by flow cytometry with an ROS assay kit (Beyotime Institute of Biotechnology, Jiangsu, China) as described previously (Zhao et al., 2018).

### Quantitative Real-Time Reverse Transcription PCR (qRT-PCR)

RAW264.7 cells were treated with RANKL (50 ng/ml) for 3 days, qRT-PCR was performed as we described previously (Zhao et al., 2018). Briefly, Total cellular RNA from cultured RAW264.7 cells was isolated with TRIzol reagents (Invitrogen, Carlsbad, CA, United States). First-strand cDNA was synthesized from 2 μg of total RNA with MMLV reverse transcriptase (Promega, Madison, WI, United States). Templates were amplified with the SYBR Green Master Mix (Invitrogen China Limited) on the iCycler real time PCR instrument (Bio-Rad, CA, United States). Primers synthesized by Invitrogen were as follows (sequences 5′ to 3′, sense and antisense): *Rank*: CAGGAGAGGCATTATGAGCA and GGTACTTTCCTGGTTCGCAT; *Trap*: GATGCCAGCGACAAGAGGTT and CATACCAGGGGATGTTGCGAA; *Cathepsin K*: GAAGAAGACTCACCAGAAGCAG and TCCAGGTTATGGGCAGAGATT; matrix metalloproteinase-9 (*Mmp-9*): CTGGACAGCCAGACACTAAAG and CTCGCGGCAAGTCTTCAGAG; *C-Fos*: GGTGAAGAGCCGTGTCAGGAG and TATTCCGTTCCCTTCGGATT; *Nfatc1*: CAACGCCCTGACCACCGATAG and GGGAAGTCAGAAGTGGGTGGA; GAPDH: ACCCAGAAGACTGTGGATGG and CACATTGGGGGTAG GAACAC.

### Western Blot Analysis

Immunoblot analysis was done in RAW264.7 cells as described earlier (Guan et al., 2013, 2015). The primary antibodies included mouse anti-GAPDH and anti-β-Actin were from Boster (Wuhan, China). Cells were lysed using the protein extraction reagent RIPA (Boster, Wuhan, China) supplement with 1 mM PMSF. The protein concentration was determined using the BCA assay. An equivalent amount of protein was resolved by 10% SDS-PAGE gel and transferred to PVDF membranes (Millipore, Billerica, MA, United States). Subsequently, membranes were blocked and immunoblotted with individual antibodies. The membranes were washed and incubated with horseradish peroxidase-conjugated secondary antibodies (Boster, Wuhan, China). The immunoreactive proteins were visualized using enhanced chemiluminescence (Boster, Wuhan, China) and captured by a scanner (ChemiDoc MP, Bio-Rad, United States).

### Electrophoretic Mobility Shift Assay (EMSA)

Electrophoretic mobility shift assay was performed as described previously (Guan et al., 2013; Zhao et al., 2017). NF-κB and AP-1 DNA-binding activity was detected using a LightShift Chemiluminescent EMSA Kit (Thermo Fisher Scientific, China). RAW264.7 cells were pretreated with taxifolin with a concentration of 100 μM for 2 h and subsequently stimulated with 50 ng/ml RANKL for 30 min. Nuclear extracts were prepared with Nuclear and Cytoplasmic Protein Extraction Kit (Beyotime Institute of Biotechnology, Jiangsu, China). With help of LightShift Chemiluminescent EMSA Kit (Thermo Fisher Scientific, China), an equal amount of nuclear extracts was incubated with biotin end-labeled duplex DNA and electrophoresed on a 6% polyacrylamide native gel. AP-1 and NF-κB probes (Beyotime Institute of Biotechnology, Jiangsu, China) used for EMSA containing the consensus recognition sites were as follows: NF-κB, 5′-AGTTGAGGGGACTTTCCCAGGC-3′; AP-1, 5′-CGCTTGATGACTCAGCCGGAA-3′.

### Statistical Analysis

Experiments were done at least three times with similar results. Data were expressed as mean ± SD. Student’s *t*-test was used for comparison between two groups. In case of comparison involving more than two groups, Analysis of Variance (ANOVA) was used. Statistical significance was considered as ^∗^*P* < 0.05, ^∗∗^*P* < 0.01, ^∗∗∗^*P* < 0.001.

## Results

### Bone Loss and Osteoclast Activity in OVX Mice

**FIGURE 1:**
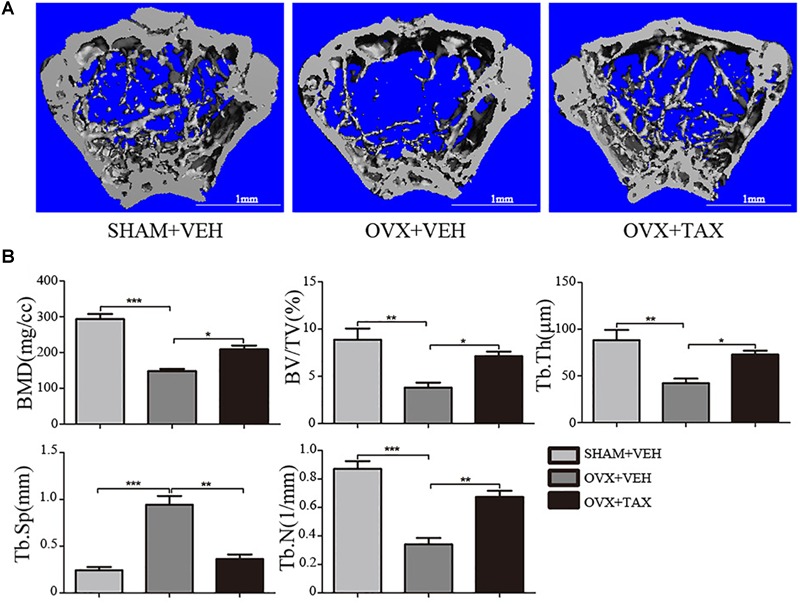
Taxifolin prevents bone loss in ovariectomized mouse. **(A)** Micro-CT images of trabecular bone near the distal femoral metaphyseal region from representative specimens of Sham+VEH (vehicle-treated sham-operated controls), OVX+VEH (vehicle-treated ovariectomized mice), and OVX+taxifolin (taxifolin-treated ovariectomized mice). Six weeks after operation, 3D trabecular architecture was studied using a μCT. Scale bars, 1 mm. **(B)** Bone mineral density (BMD) Bone value/total value (BV/TV), trabecular number (Tb.N), trabecular space (Tb.Sp), and trabecular thickness (Tb.Th) were analyzed with the built-in software of the μCT as described in the Section “Materials and Methods” (^∗^*P* < 0.05, ^∗∗^*P* < 0.01, ^∗∗∗^*P* < 0.001 versus OVX group, *n* = 12).

**FIGURE 2:**
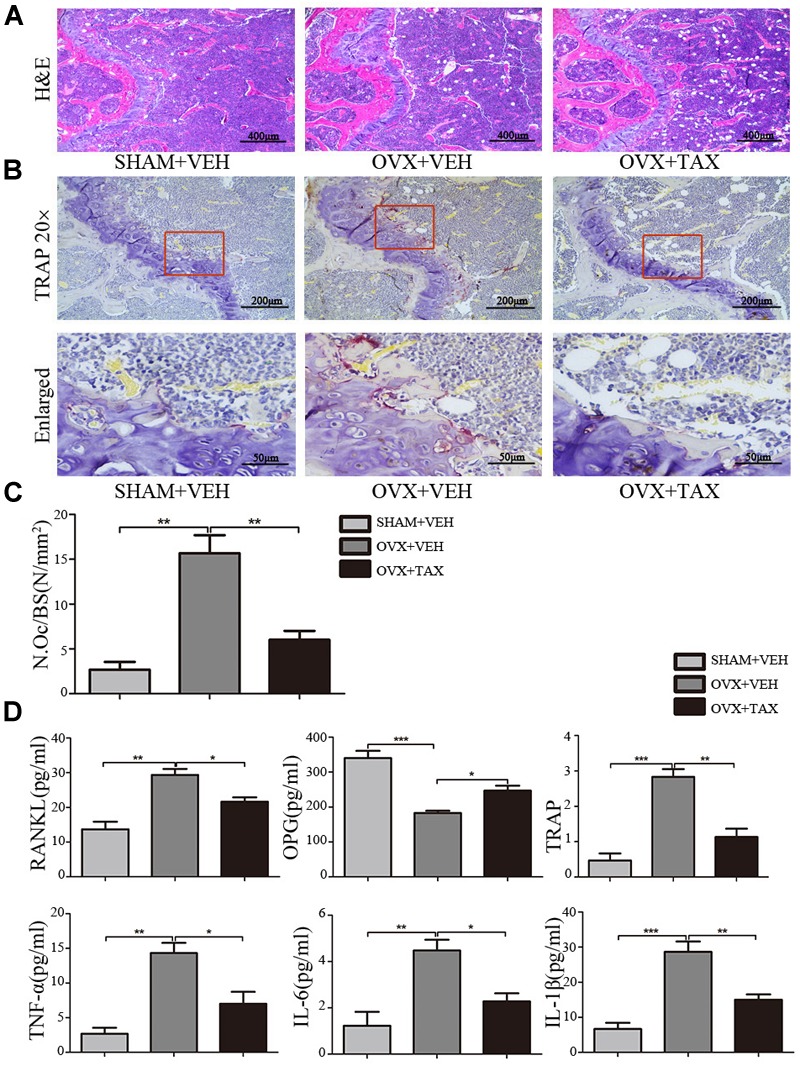
Taxifolin decreases bone resorption and osteoclast formation in OVX mice. **(A)** Representative hematoxylin and eosin (H&E) staining of femoral sections from each group 6 weeks after operation. Scale bars, 400 μm. **(B)** Representative TRAP-stained histologic femur sections of long bone from sham, OVX mice and OVX+ taxifolin mice for visualization of the red-colored TRAP+ osteoclasts. 20× scale bars, 200 μm; enlarged scale bars, 50 μm. **(C)** The osteoclast number/bone surface was quantified. **(D)** Serum levels of OPG, RANKL, TRAP, IL-6, IL-1β, and TNF-α were determined by ELISA (*n* = 12 per group, ^∗^*P* < 0.05, ^∗∗^*P* < 0.01, ^∗∗∗^*P* < 0.001).

We evaluated the effects of taxifolin on bone loss using an ovariectomized mouse model. As expected, 6 weeks after operation (Figure 1), OVX mice exhibited a significant loss of trabecular bone, as revealed by decreased BMD, trabecular bone volume (BV/TV), trabecular thickness (Tb.Th) and trabecular numbers (Tb.N), and by increased trabecular space (Tb.Sp), compared to the sham-operated mice. Meanwhile, treatment with taxifolin in OVX mice dramatically attenuated trabecular bone loss, as shown by the histomorphometry parameters in comparison to OVX mice treated with vehicle. The results were further corroborated by decalcified H&E stained bone sections (Figure 2A). Femoral sections from OVX mice demonstrated a paucity of cancellous bone both proximal and distal to the growth plate. The trabeculae in both regions is scarce and thin. Taxifolin treatment in the OVX mice induced a marked increase in bone density, with significant increase in trabecular thickness and density compared with the OVX mice treated with vehicle. These sections of the OVX+taxifolin group were practically indistinguishable from sections of the Sham+VEH group.

We next examined osteoclast differentiation in OVX mice treated with taxifolin. Compared with OVX mice treated with vehicle, mice with taxifolin treatment displayed much less red-colored TRAP-positive multinucleated cells at the growth plates of the long bones (Figure 2B), reduced osteoclast numbers per bone surface (N.Oc/BS) (Figure 2C), decreased serum TRAP levels, a serologic marker of osteoclast function (Figure 2D). These observations suggest that taxifolin function as a strong inhibitor of osteoclastogenesis and resorption activity. Taxifolin also effectively decreased serum levels of TNF-α, IL-6, IL-1β, and RANKL and increased the serum level of OPG (Figure 2D).

### Osteoclast Differentiation and Activity *in vitro*

**FIGURE 3:**
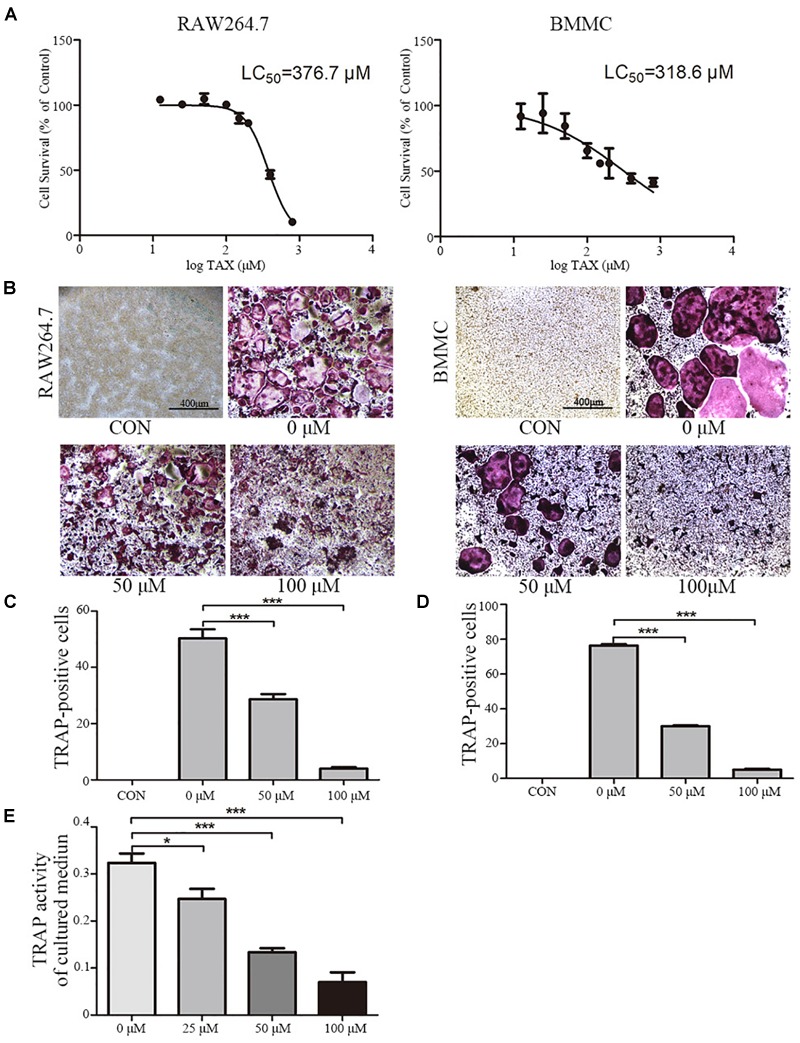
Taxifolin inhibits osteoclast differentiation and activity *in vitro*. **(A)** RAW264.7 and BMMCs (cultured with M-CSF at 25 ng/ml) were treated with taxifolin of different concentrations. After 3 days, an LC_50_ curve was used to measure cytotoxicity. **(B–D)** RAW264.7 and BMMCs (cultured with M-CSF at 25 ng/ml) were treated with RANKL (50 ng/ml) and taxifolin of different concentrations for 4 days as indicated in the figures, cells were used for TRAP staining and TRAP enzyme activity assay. TRAP+ cells with three or more nuclei were identified as osteoclasts. **(B)** Taxifolin inhibits osteoclast formation in a dose-dependent manner. Scale bars, 400 μm. **(C,D)** Quantitation of osteoclasts formed by RAW264.7 and BMMCs. **(E)** TRAP activity in cultured medium of BMMCs was measured. Data are presented as mean ± SD. *n* = 3, ^∗^*P* < 0.05, ^∗∗^*P* < 0.01, ^∗∗∗^*P* < 0.001.

To further explore roles played by taxifolin in osteoclastogenesis, we next validated its effects on RAW264.7 cells and BMMCs. LC_50_ curve was calculated by GraphPad Prism 5 according to MTT cytotoxicity assay results to measure cytotoxicity (Figure 3A). Taxifolin treatment significantly reduced numbers of the TRAP-positive multinuclear cells in a dose-dependent manner (Figures 3B–D), with the maximal effect at 100 μM of concentration. Similarly, the TRAP enzyme activity in culture medium was decreased by taxifolin treatment (Figure 3E).

### Osteoclast Activity

**FIGURE 4:**
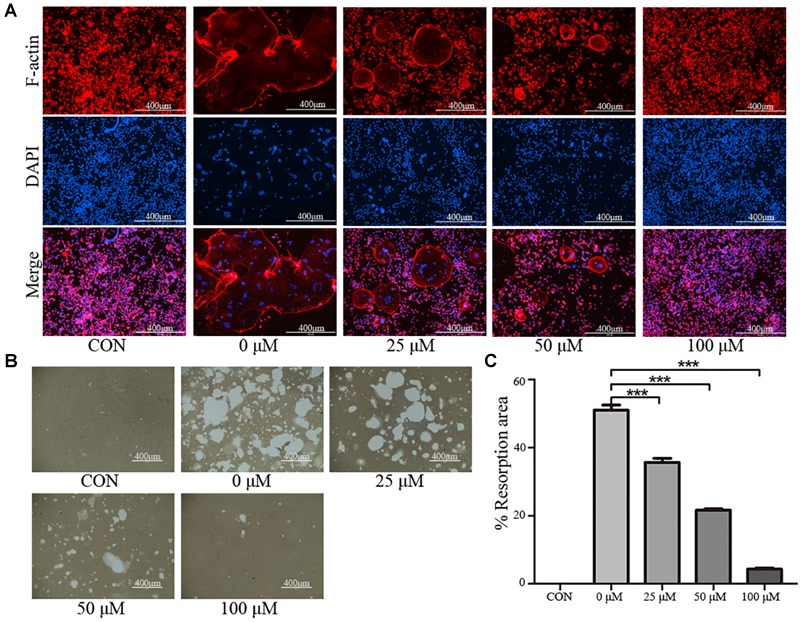
Taxifolin inhibits osteoclast function. **(A)** Taxifolin disrupted the actin ring formation. RAW264.7 cells were treated with RANKL and with or without different concentrations of taxifolin, after 4 days, actin ring formation staining was performed and subsequently examined by fluorescence microscopy. Scale bars, 400 μm. **(B,C)** Taxifolin inhibited osteoclast bone resorption function. Mature osteoclasts were collected and seeded onto a Corning Osteo Assay Surface and treated with or without different concentrations of taxifolin for 3 days. Images were taken and resorption was quantified by image analysis. Scale bars, 400 μm. Data are presented as mean ± SD. *n* = 3. ^∗^*P* < 0.05, ^∗∗^*P* < 0.01, ^∗∗∗^*P* < 0.001.

To further examine the effects of taxifolin on osteoclast activity, actin ring formation assays and bone slice resorption assays were performed. The results indicated that actin ring formation was inhibited by taxifolin treatment (Figure 4A). Formed osteoclasts were lifted, seeded on bone slice, and then treated with different concentrations of taxifolin. Taxifolin potently suppressed the bone resorption activity of osteoclasts (Figures 4B,C). These findings indicated that taxifolin impairs the function of mature osteoclasts.

### Expression of Multiple Osteoclast Specific Genes and Proteins

**FIGURE 5:**
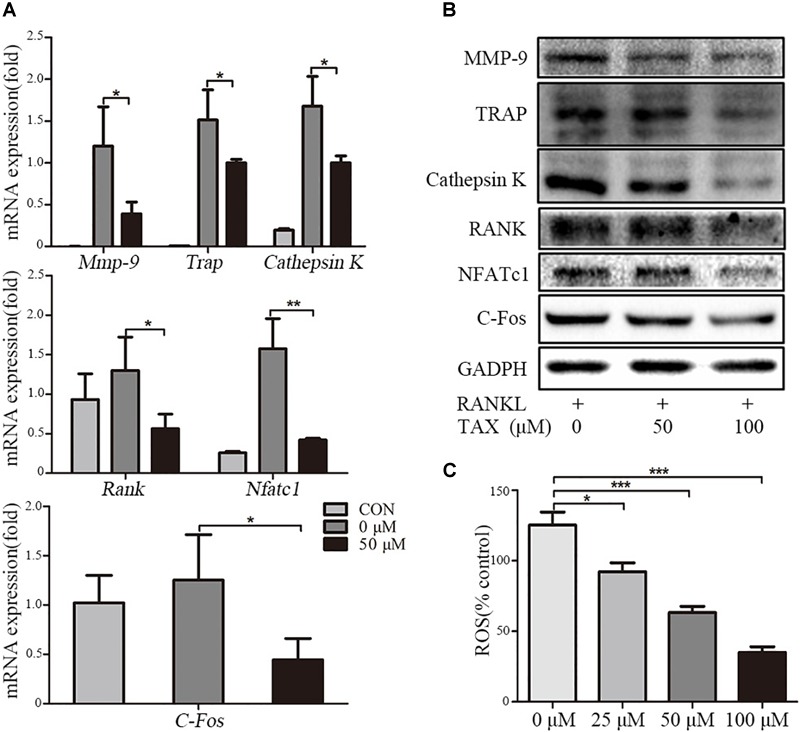
Taxifolin suppresses expression of osteoclast specific genes and proteins. RAW264.7 cells were treated with RANKL and with or without different concentrations of taxifolin for 3 days, **(A)** expression of *Trap, Mmp-9, Cathepsin K, C-Fos, Nfatc1*, and *Rank* was determined by qRT-PCR and calculated in relation to the internal control GAPDH mRNA by the comparative Ct method; **(B)** immunoblots with MMP-9, TRAP, Cathepsin K, RANK, NFATc1 and C-Fos antibodies demonstrating that taxifolin repressed osteoclast-specific protein expression. GAPDH antibody was used as loading controls. **(C)** RAW264.7 cells were cultured with taxifolin for 36 h, then stimulated with RANKL (50 ng/ml) for 30 min, and RAW264.7 cells without RANKL or taxifolin was considered as “100% control,” our results showed taxifolin decreased the release of intracellular ROS. Data are presented as mean ± SD. *n* = 3, ^∗^*P* < 0.05, ^∗∗^*P* < 0.01, ^∗∗∗^*P* < 0.001.

To investigate the mechanisms of inhibited osteoclastogenesis from RAW264.7 cells by taxifolin, we measured the expression of osteoclast specific genes after taxifolin treatment. In osteoclasts generated from RAW264.7 cells, pretreatment with taxifolin significantly inhibited mRNA expression of *Trap, Mmp-9, Cathepsin K, C-Fos, Nfatc1*, and *Rank* (Figure 5A). Western blots using antibodies against MMP-9, TRAP, Cathepsin K, RANK, NFATc1 and C-Fos also demonstrated the downregulation of these osteoclast specific proteins by taxifolin from RAW264.7 cells (Figure 5B). The inhibition of the expression of osteoclast markers by Taxifolin might be due to suppressing RANKL signaling.

### The Release of Intracellular ROS

RANKL stimulation increases the intracellular level of ROS, and ROS also activates osteoclast differentiation. We measured the release of intracellular ROS, our results showed that the production of ROS was increased through RANKL stimulation, but it was effectively decreased by taxifolin in a dose-dependent manner (Figure 5C).

### Pathways Involved in Osteoclast Differentiation

**FIGURE 6:**
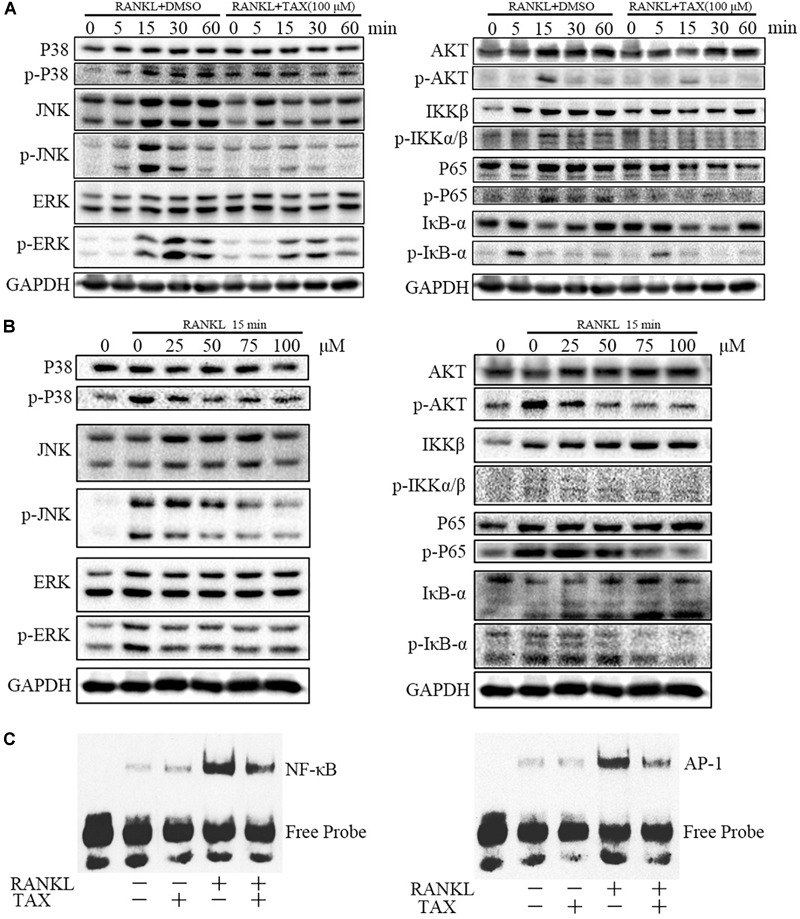
Taxifolin represses multiple pathways of osteoclastogenesis in RAW264.7 cells. **(A)** RAW264.7 cells were pre-treated with 100 μM taxifolin for 2 h, then stimulated with RANKL (50 ng/ml) for the indicated time, protein was extracted for immunoblotting, the same GAPDH was used as loading control. **(B)** RAW264.7 cells were pre-treated with taxifolin in indicating concentrations for 2 h, then stimulated with RANKL (50 ng/ml) for 15 min, protein was extracted for immunoblotting, the same GAPDH was used as loading control. **(C)** Electrophoretic mobility shift assay for DNA binding activity of NF-κB and AP-1. After treatment with 100 μM taxifolin for 2 h, RAW264.7 cells were stimulated with RANKL (50 ng/ml) for 30 min, then nuclear extracts were prepared and analyzed for DNA binding activity. Data are of three independent experiments.

We further measured the activity of major molecular pathways in taxifolin treated RAW264.7 cells (Figures 6A,B). Among the three major subfamilies of MAPK, levels of the phosphorylated p38 (p-P38), phosphorylated ERK (p-ERK), and phosphorylated JNK (p-JNK) was all showing pronounced increase upon RANKL stimulation and taxifolin treatment completely prevented their response to RANKL in a dose-dependent and time-dependent manner. Furthermore, taxifolin also inhibited the levels of AKT phosphorylation (p-AKT), IKKβ phosphorylation (p- IKKβ), IkBα phosphorylation (p-IkBα), and p65 phosphorylation (p-P65).

### DNA Binding Activity of NF-κB and AP-1

Transcription factors such as NF-κB and AP-1 are known to play an essential role in osteoclastogenesis. As shown (Figure 6C), 30 min after stimulation with RANKL, the DNA-binding activity of the transcription factors NF-κB and AP-1 increased pronouncedly. Taxifolin alone had no significant influence on baseline NF-κB and AP-1 activity. Whereas, taxifolin treatment significantly impaired the activation of NF-κB and AP-1 by RANKL.

## Discussion

In the current study, we investigated the role of taxifolin in osteoclasts (Supplementary Figure 1). Our *in vivo* study demonstrated that taxifolin effectively protected against alterations in bone architecture parameters, serum bone turnover markers and the pro-inflammatory cytokines TNF-α and IL-1β. As for the molecular mechanisms, we confirmed multiple pathways, including NF-κB, MAPKs, and AKT, the downstream pathways of RANKL activated during osteoclastogenesis, were significantly inhibited by taxifolin.

Flavonoids have been proved to protect against bone loss partially through inhibiting osteoclast bone resorption, promoting osteoblast function, reducing oxidative stress or suppressing chronic low-grade inflammation (Weaver et al., 2012; Welch and Hardcastle, 2014). We investigated that NF-κB might be one of the most important pathways that taxifolin mediating osteoclastogenesis. NF-κB is a pivotal regulator of osteoclast formation and function (Takayanagi, 2007). Taxifolin has been reported to stimulate osteoblast differentiation in bone marrow mesenchymal stem cells by inhibiting TNF-α-induced NF-κB signaling pathway activation (Wang et al., 2017). Taxifolin is a kind of phytoestrogen (Jefferson et al., 2002), estrogen deficiency contributes to bone loss by increasing the production of pro-inflammatory cytokines in estrogen deficiency induced osteoporosis (Brincat et al., 2014). Several inflammatory molecules, such as TNF-α, IL-1β, and IL-6 can promote osteoclastogenesis and bone resorption indirectly by increasing expression of RANKL and M-CSF by stromal cells and T cells (Lee et al., 2010), and pro-inflammatory cytokines induced osteoclastogenesis are associated with the activation of NF-κB (Jimi et al., 2004). These hint that taxifolin may suppress osteoclastogenesis through NF-κB pathway, contrary to what we expected, our results show taxifolin inhibits multiple downstream pathways of RANK signaling, including NF-κB and MAPK. Moreover, taxifolin can reduce the serum levels of RANKL, TNF-α, IL-1β, and IL-6 in OVX-induce osteopenic mice, which reveals taxifolin can also repress the upstream pathways of RANK signaling.

Previous studies (Guo et al., 2015; Kuang et al., 2017; Xie et al., 2017) have shown taxifolin exhibits significant antioxidant properties, it can inhibit ROS production in many kinds of cells. ROS are involved in osteoclast activation and bone loss stimulated by increased expression of RANKL, ROS could act as the intracellular secondary messengers in RANKL-induced osteoclastogenesis signaling pathways, and osteoclast differentiation can be inhibited by scavenging ROS (Lee et al., 2005). We investigated the anti-oxidative activity of taxifolin on RANKL-induced osteoclast precursor-like cells and found that RANKL can increase the ROS production which can be abolished by taxifolin, these results are consistent with the previous studies. Thus, our study also suggested taxifolin alleviated bone loss *in vivo* partially by ROS signaling.

However, our *in vivo* study concentrated only to osteoclast function and ignored the bone formation by osteoblast which both contribute to the improvement bone mass in osteopenic mice. Furthermore, previous study has shown taxifolin can stimulate osteoblast differentiation in bone marrow mesenchymal stem cells (Wang et al., 2017), and regrettably, we do not clarify the mechanism of taxifolin on the potential crosstalk between osteoclast and osteoblast. Maybe, all these limitations need to be further clarified in the future studies.

Our study demonstrates that taxifolin has the promising effects on preventing bone loss and may be used as one type of estrogen replacement therapy in treating postmenopausal osteoporosis. Considering the numerous pharmacological activities of taxifolin, it is worth further study to be translated into clinical application.

## Author Contributions

LbZ and JX designed the study and interpreted the data. CC, CL, LmZ, HL, WL, HG, LbZ, and JX conducted the study. CC and LbZ collected the data. CC, HG, LbZ, and JX contributed to data analysis. CC, LbZ, and JX drafted the manuscript. All authors approved the final version of the manuscript.

## Conflict of Interest Statement

The authors declare that the research was conducted in the absence of any commercial or financial relationships that could be construed as a potential conflict of interest.
