# Supplementary material for: Effects of Taxifolin on Osteoclastogenesis in vitro and in vivo
Source: Front Pharmacol. 2018 Nov 12;9:1286. doi: 10.3389/fphar.2018.01286 (PMC6240596; doi:10.3389/fphar.2018.01286)
Supplement: Supplementary file 1 [file Data_Sheet_1.PDF]

Supplemental Figure Legends

Supplemental Figure 1

(A) Taxifolin disrupted the actin ring formation. BMMCs (cultured with M-CSF at 25 ng/ml) were treated with RANKL and with or without different concentrations of taxifolin, after 4 days, actin ring formation staining was performed and subsequently examined by fluorescence microscopy. Scale bars, 400 μm. (B) Taxifolin inhibited osteoclast bone resorption function. Mature osteoclasts were collected and seeded onto a Corning Osteo Assay Surface and treated with or without different concentrations of taxifolin for 3 days. Images were taken and resorption was quantified by image analysis. Scale bars, 400 μm. Data are presented as mean ±SD. n = 3. *P < 0.05, **P < 0.01, ***P < 0.001.
