# Supplementary figures and images for: Effects of Taxifolin on Osteoclastogenesis in vitro and in vivo
Source: Front Pharmacol. 2018 Nov 12;9:1286. doi: 10.3389/fphar.2018.01286 (PMC6240596; doi:10.3389/fphar.2018.01286)

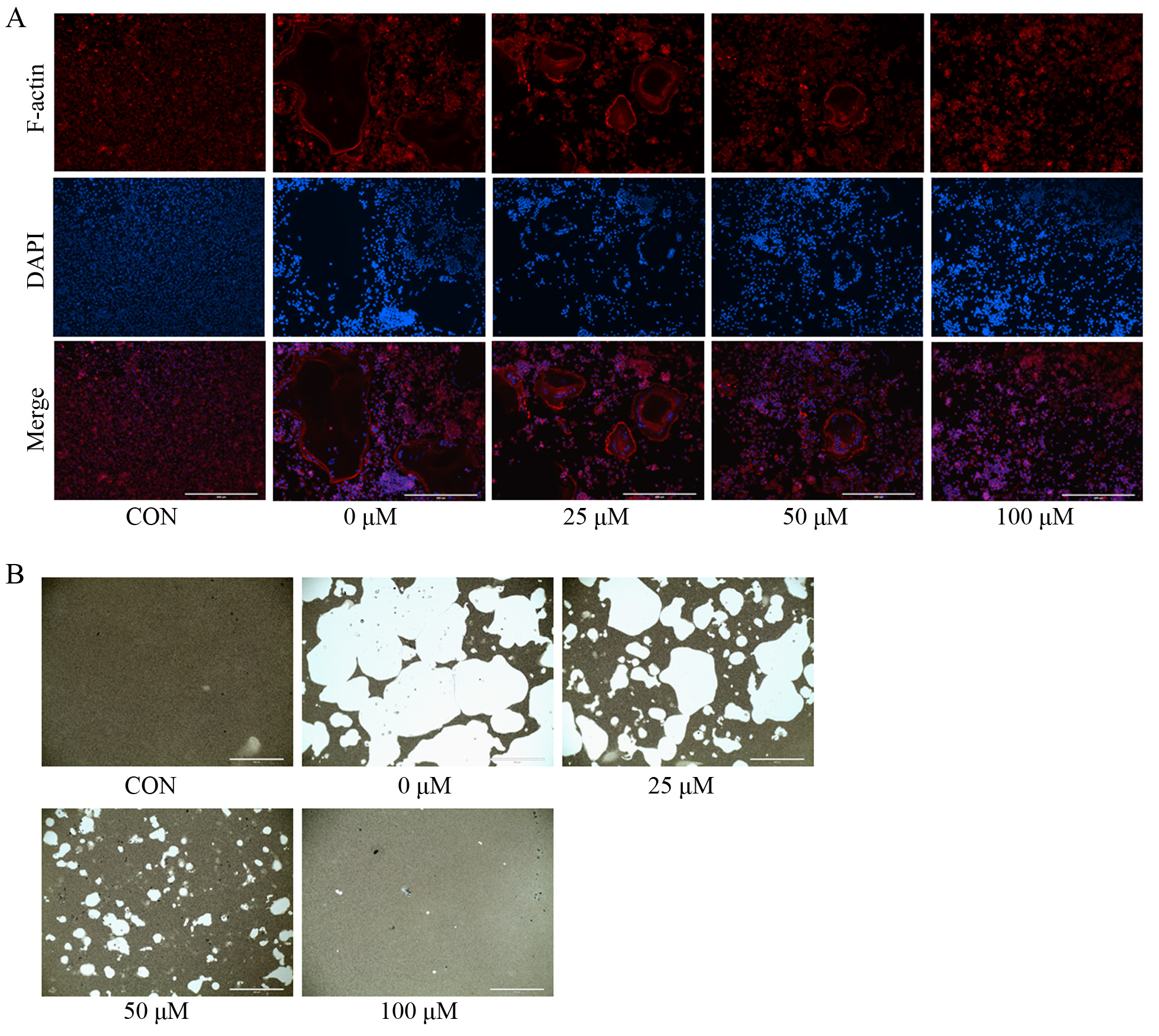

Supplement: Supplementary file 2 [file Image_1.JPEG]
